# Single Nucleotide Polymorphism Highlighted via Heterogeneous Light-Induced Dissipative Structure

**DOI:** 10.1021/acssensors.4c02119

**Published:** 2025-01-23

**Authors:** Shuichi Toyouchi, Seiya Oomachi, Ryoma Hasegawa, Kota Hayashi, Yumiko Takagi, Mamoru Tamura, Shiho Tokonami, Takuya Iida

**Affiliations:** † Research Institute for Light-induced Acceleration System (RILACS), Osaka Metropolitan University, 1-2 Gakuencho, Nakaku, Sakai, Osaka 599-8570, Japan; ‡ Department of Physics, Graduate School of Science, Osaka Metropolitan University, 1-2 Gakuencho, Nakaku, Sakai, Osaka 599-8570, Japan; § Department of Materials Science, Graduate School of Engineering, Osaka Metropolitan University, 1-2 Gakuencho, Nakaku, Sakai, Osaka 599-8570, Japan; ∥ Department of Materials Engineering Science, Graduate School of Engineering Science, Osaka University, 1-3 Machikaneyama-cho, Toyonaka, Osaka 560-8531, Japan

**Keywords:** single nucleotide polymorphisms, fluorescence, nanoparticles, optical condensation, optical force, photothermal effect

## Abstract

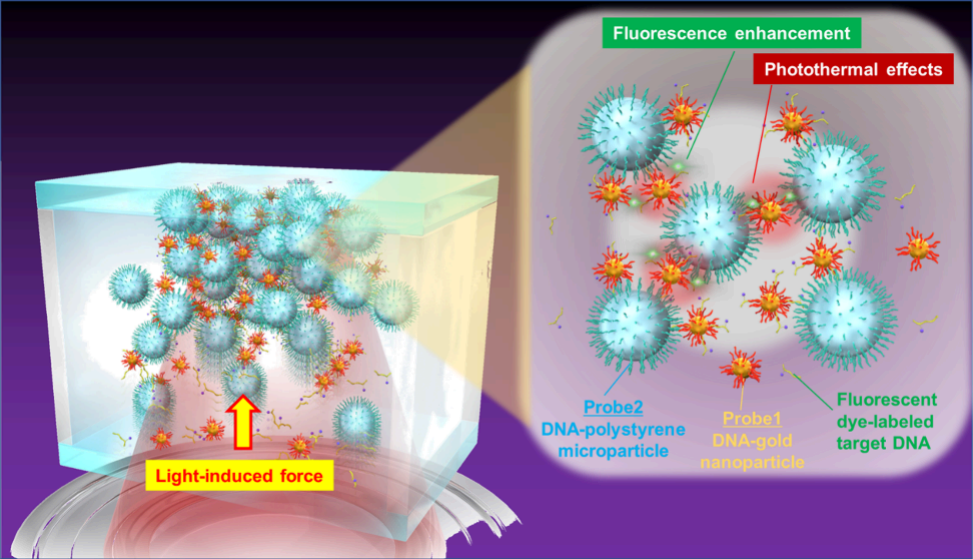

The unique characteristics of biological structures depend on the behavior of DNA sequences confined in a microscale cell under environmental fluctuations and dissipation. Here, we report a prominent difference in fluorescence from dye-modified single-stranded DNA in a light-induced assembly of DNA-functionalized heterogeneous probe particles in a microwell of several microliters in volume. Strong optical forces from the Mie scattering of microparticles accelerated hybridization, and the photothermal effect from the localized surface plasmons in gold nanoparticles enhanced specificity to reduce the fluorescence intensity of dye-modified DNA to a few %, even in a one-base mismatched sequence, enabling us to clearly highlight the single nucleotide polymorphisms in DNA. Fluorescence intensity was positively correlated with complementary DNA concentrations ranging in several tens fg/μL after only 5 min of laser irradiation. Remarkably, a total amount of DNA in an optically assembled structure of heterogeneous probe particles was estimated between 2.36 ymol (2.36 × 10^–24^ mol) and 2.36 amol (2.36 × 10^–18^ mol) in the observed concentration range. These findings can promote an innovative production method of nanocomposite structures via biological molecules and biological sensing with simple strategies avoiding genetic amplification in a PCR-free manner.

Thermal fluctuations and energy dissipation are inextricably linked in the formation of dissipative structures under nonequilibrium conditions and are especially important in the formation of ordered biological structures.^1^ Since the inception of life on earth 3.5 billion years ago, optimized photoreceptors, photosynthetic antennae, and ordered structures with diverse spatial patterns have evolved under various external fields such as thermal fluctuations and ambient light.^2−4^ Over the course of evolution, DNA formed a narrow space enclosed by a lipid bilayer in which information was transcribed by RNA, a system for protein production was established, and self-replication was achieved in accordance with the central dogma.^5,6^ The double-helix structure of DNA elucidated in 1953,^7^ and the base sequence of human DNA reported by the Human Genome Project^8^ represent notable milestones in the life sciences. However, the mechanisms underlying the precise transmission of genetic information and mutations in various clinically important molecules, such as KRAS and EGFR in cancer development, remain unclear.^9,10^ Recent theories indicate that quantum tunneling plays important roles in gene mutations, and the quantum nature of hydrogen bonding influences the formation of ordered structures in vivo.^11,12^

Evaluation of environmental DNA^13^ is important to understand ecosystem transitions and environmental changes under climate change. Cell-free DNA (cfDNA)^14,15^ and circulating tumor DNA (ctDNA)^16^ have also attracted attention as important biomarkers in liquid biopsies used in the diagnosis of genetic diseases, such as cancer. In those emerging DNA sensing applications, single-nucleotide polymorphisms (SNPs) are required to be exclusively detected from wild genes or other species. Thus, simple, high-throughput genetic, and highly gene-specific measurement techniques are essential for both environmental conservation and clinical care. Many techniques have been developed to analyze the differences and mutations in DNA sequences, including the Sanger sequencing using fluorescence and electrophoresis^17^ and polymerase chain reaction (PCR) based on primer-based amplification.^18^ Furthermore, next-generation sequencing, which incorporates the advantages of Sanger sequencing and PCR, has enabled high-throughput analysis^19^ of genetic changes in cancer, dementia, and infectious diseases, making their early diagnosis more accessible. Since the discovery of PCR in the early 1980s, PCR-based methodologies have revolutionized gene diagnostics and are currently considered the gold standard for gene analysis. However, PCR-based gene analysis is expensive, time-consuming, and requires complex instrumentation, qualified personnel, and specialized laboratories. Recent progress in next-generation sequencing has enabled the simultaneous determination of sequences of thousands to millions of DNA molecules; however, the required equipment is large and expensive, and the entire process takes hours to days. Therefore, more accessible and user-friendly technologies are needed to overcome the limitations of PCR-based methods and advance the current paradigm of diagnostics. PCR-free strategies represent promising solutions for gene analysis without the amplification step, which can substantially reduce costs and improve sequencing turnover.

In genetic sequencing, target biological materials should ideally be transported toward the observation region of a sensor in a simple and effective manner. Based on the optical tweezers proposed by Ashkin et al.,^20^ tiny biological and nonliving materials can be precisely manipulated through electromagnetic interactions driven by light-induced force (LIF). This technology can nondestructively capture and extend a long DNA strand by trapping and moving a microparticle linked to the DNA.^21^ Optical manipulation technology has been widely applied in nanoscale physics, chemistry, and biology.^22−24^ However, the working range of optical tweezers is limited to the laser-irradiated area. In contrast, by optically trapping metal nanostructures with localized surface plasmons, light-induced convection (LIC) is generated over the liquid sample through photothermal effects, thereby guiding and condensing dispersoids toward the observation region^25^ where the synergistic effects of the LIF and LIC facilitate the optical condensation and reaction of small amounts of biological nanomaterials.^26^ With specific surface-structure substrate designs, micron-scale biological objects such as bacteria can be optically condensed with a high survival rate, even by LIC arising from the photothermal effect.^27,28^ Furthermore, optical condensation through light-induced acceleration of selective antigen–antibody reactions and cellular uptake can be controlled by balancing the LIF and LIC.^29−32^ Paying attention to previous studies on material engineering with biological molecules, there are many reports on the construction of various assembled structures consisting of nanoparticles (NPs) via DNA hybridization,^33−35^ and on the electric and nanophotonic biosensors using molecular recognition.^36,37^ For example, DNA-modified gold NPs have been used for the electric detection of target DNA at 100 pmol/μL.^38^ The hybridization of DNA-modified NPs and floating single-stranded DNA is accelerated by LIF mediated by LIC at the air–liquid interface.^39^ Nonresonant laser irradiation is associated with little light-induced polarization and weak LIFs on NPs smaller than the light wavelength,^40^ thus requiring the assistance of LIC with evaporation. The application of optical condensation is further limited by the large size variation in the assembled composites and requires local condensation at the solid–liquid interface for the stable construction of DNA-modified composite NPs.

Here, based on the light-induced acceleration system for molecular recognition, we aimed to develop a new macroscopic light-induced assembly (LIA) method for DNA-modified composites based on two types of probe particles (Figure 1A, B). In this process, DNA molecules are modified on microparticles (probe 1), on which a strong LIF acts through Mie scattering, and gold NPs (probe 2), which enhance the electric field and the photothermal effect of localized surface plasmons. We attempted to enhance LIC and LIF via the assembly of low-density NPs and target substances by capitalizing on the collective micron-scale phenomena of localized surface plasmons. In a static fluid in a microwell, probe particles were assembled at the solid–liquid interface by laser irradiation, and the target DNA was condensed by hybridization among the heterogeneous probe particles in a sequence-specific manner. A fluorescent dye modification was used to observe the target DNA trapped in the LIA via fluorescence imaging. Finally, we evaluated the suitability of our approach for quantitatively detecting target DNA at low concentrations.

**Figure 1:**
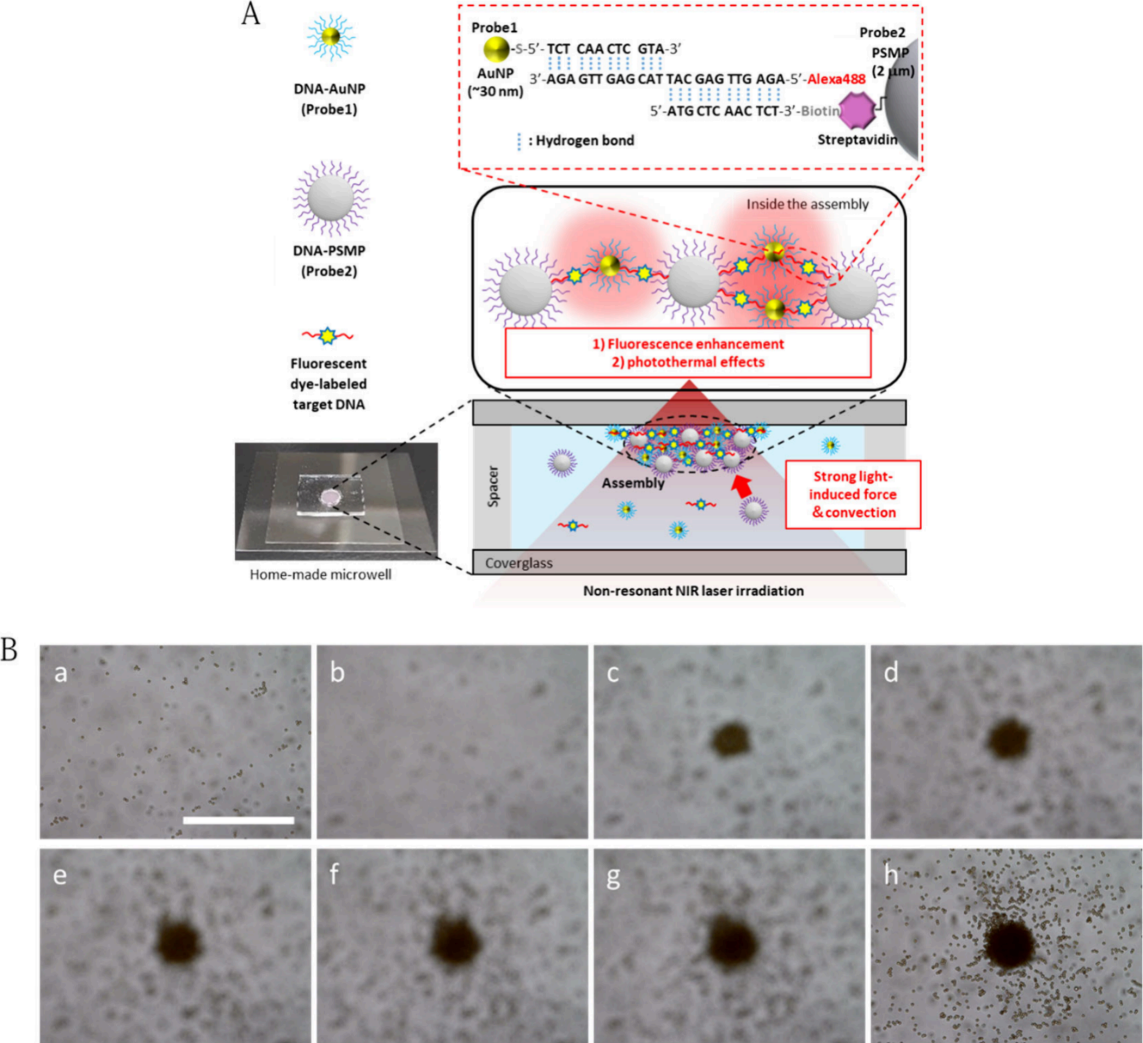
Light-induced acceleration of DNA hybridization with optically assembled DNA-functionalized gold nanoparticles (DNA-AuNP, Probe 1) and polystyrene microparticles (DNA-PSMP, Probe 2) as heterogeneous probe particles for PCR-free DNA detection. (A) Schematic illustration of experimental design. An infrared laser was loosely focused near the glass–liquid interface of a homemade microwell, where probe particles and fluorescent dye-labeled target DNA molecules were transported by light-induced force (LIF) and light-induced convection (LIC) flow and then assembled. Inside the light-induced assembly (LIA), the complementary target DNA molecules bridge the heterogeneous probes by forming double-stranded DNA. AuNPs facilitate sequence-specific DNA detection by enhancing the fluorescence signal of target DNA through surface plasmon resonance and increasing the temperature inside the LIA through excellent photothermal effects. (B) Transmission images recorded ‘a’ before laser irradiation, during (b; 0 s, c; 1 min, d; 2 min, e; 3 min, f, 4 min, g; 5 min), and after the optical condensation of heterogeneous probe particles and target DNA. Images b-g were recorded by intentionally shifting the focal plane above 30 μm from the coverglass–liquid interface to expand the laser spot about 30 μm. In h, the focal plane moved to the ceiling of the microwell after laser irradiation. Scale bar: 100 μm. See also Movie S1.

## Results and Discussion

### Light-Induced Acceleration of DNA Hybridization

A mixture of DNA-modified gold nanoparticle (AuNP) Probe <I> and DNA-modified polystyrene microparticle (PSMP) Probe <IV> dispersion liquid (heterogeneous probe-particles) and target-DNA solution (7.37 pg/μL) was laser-irradiated in a homemade microwell for 5 min. For details on the probe preparations and the experiment, see the Methods. In this study, we prepared four DNA-modified probes; the DNA-modified AuNP probe <I> and <II> and the DNA-modified PSMP probe <II> and <IV>. The probe DNA sequences and the probe combinations are described in Table S1. The target DNA sequences are described in Table S2. Optical transmission images and the movie captured during laser irradiation are demonstrated in Figure 1B and Movie S1, respectively. For the transmission imaging during laser irradiation, the focal plane was intentionally shifted 30 μm above to expand the laser spot about 30 μm. We observed PSMP movement owing to LIFs (mainly the scattering force) and the gradual assembly of particles in the laser-irradiated area. The strength of LIFs exerted on the PSMPs and pushing them toward the laser-irradiation area is related to several experimental conditions, i.e. laser power density, laser wavelength, particle material (refractive index and extinction cross-section), and particle size. We estimated the strength was 36.4 pN at the ceiling coverglass of the homemade microwell (the estimation method described in the previous literatures^29,40^). Notably, probe particles were assembled in this area, and several particles were ejected to the outside of the laser-irradiation cone. This indicated that the LIFs exerted on the microparticles and the motion of the microparticles toward the laser irradiation area resulted in convection in the static fluid. Such a wide range LIC was theoretically and experimentally studied in the photothermal optical condensation of AuNPs and bacteria^26,28^ and in the nonthermal optical trapping of PSMPs.^41^ Because the AuNPs (d = 30 nm) and target DNA molecules were quite small, the exerted LIFs were almost negligible. However, it is assumed that the LIC may facilitate the transport of AuNPs and target DNA molecules toward the laser-irradiated area, potentially promoting their condensation within the assembly.

**Figure 2:**
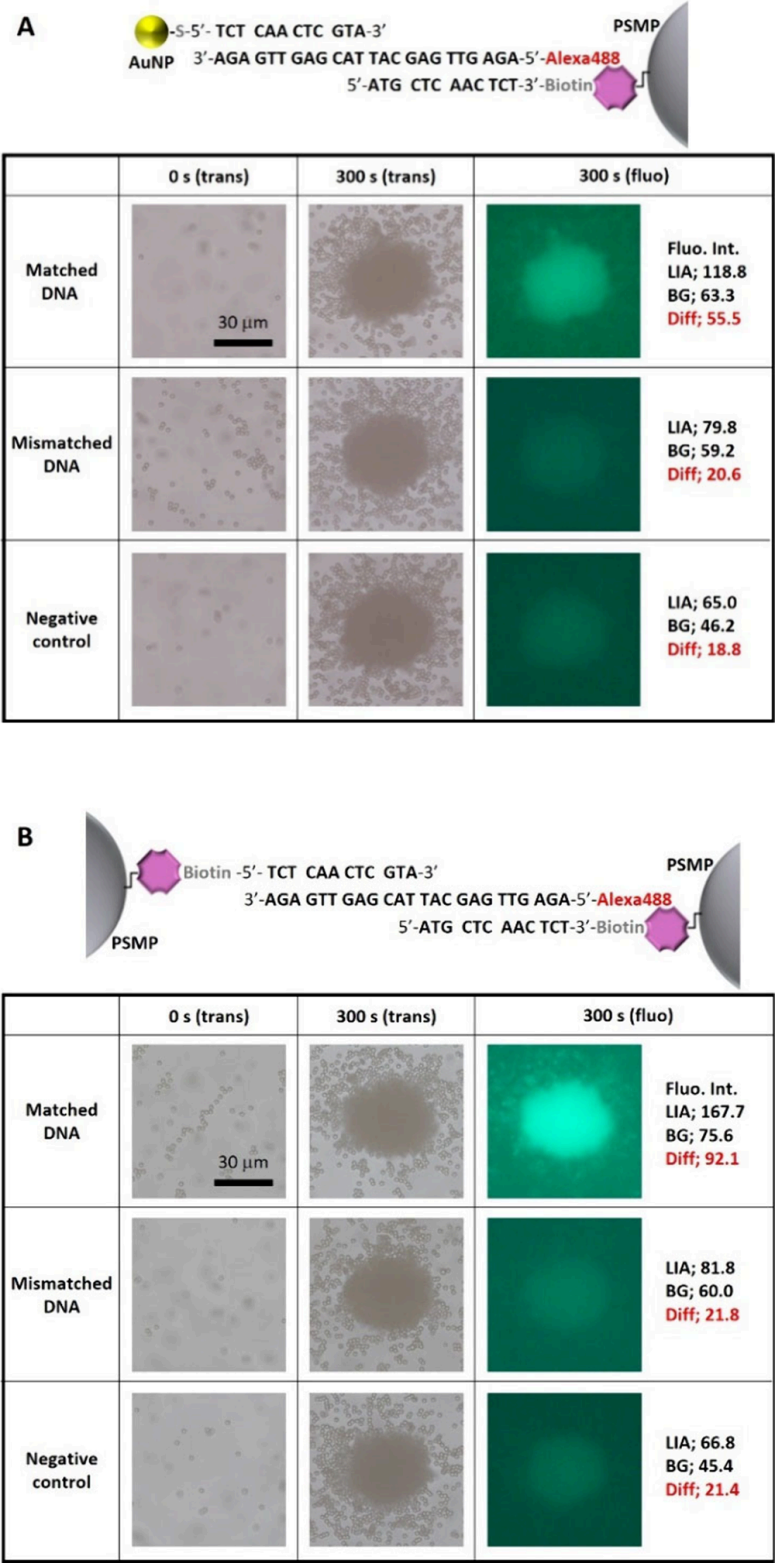
Light-induced acceleration of DNA hybridization. (A) The LIA of target DNA and heterogeneous probe particle (top) was monitored by transmission imaging using a CMOS camera before (0 s, left) and after 1064 nm continuous wave laser irradiation for 300 s (middle). Fluorescence images were captured after laser irradiation (right). The target DNA was complementary to the probe DNA sequence (Matched, 7.37 pg/μL, upper row) or fully mismatched DNA (Mismatched, 7.37 pg/μL, middle row). A negative control (target DNA 0 pg/μL) was examined under the same experimental conditions. (B) The same light-induced acceleration experiment (as in A) performed with the homogeneous probes. Fluorescence intensity (Fluo. Int.) at the LIA and background (BG) as well as the difference (Diff) between them are listed on the right side.

Figure 2A shows the typical transmission images recorded before and after 5 min laser irradiation and typical fluorescence images recorded after laser irradiation with heterogeneous probe particles. The upper, middle, and lower sections show the results with matched DNA (complementary to the probe DNA described in Table S2), mismatched DNA (perfectly mismatched to probe DNA described in Table S2), or phosphate buffer solution as a negative control (NC, without any target DNA), respectively. Regardless of the presence or absence of DNA and sequence matches or mismatches, LIA was observed, with sizes almost comparable to those of the laser-irradiated area. A remarkable difference was observed in the fluorescence measurements. For matched DNA, the fluorescence intensity (defined as the difference between the averaged brightness in the LIA and background areas) was ∼55.5. In contrast, the fluorescence intensity was ∼20.6 for the mismatched DNA and ∼18.8 for the NC, which was comparable to that of the mismatched DNA but significantly weaker than that of the matched DNA. These results indicated that the target DNA molecules were selectively trapped and condensed inside the LIA through DNA hybridization. Note that a spontaneous probe assembly and DNA localization were not observed at the laser-irradiation area in the homemade microwell without laser irradiation, even after several hours. Although a spontaneous probe assembly was observed for an unsealed mixture droplet on a coverglass, it took more than 20 min even for 7.37 ng/μL of target DNA (see Figure S1). Therefore, compared with the spontaneous probe assembly, we concluded that merely 5 min of laser irradiation condensed the DNA molecules in the LIA, and accelerated DNA hybridization by the optical condensation.

We examined the optical condensation of homogeneous probe particles, comprising a mixture of two DNA-modified PSMP Probe <III> and <IV> dispersion liquids, for comparison with the heterogeneous probe particles. Figure 2B shows the typical transmission and fluorescence images of the homogeneous probe-particles. Similar to the heterogeneous probe particles, we observed LIA in the laser irradiation area regardless of match DNA, mismatch DNA, or NC. A remarkable difference in fluorescence intensity was observed between the matched DNA (∼92.1), mismatched DNA (∼21.8), and NC (∼21.4) (Figure 2B). The fluorescence intensities were comparable between mismatched DNA (Figure 2B middle) and NC (Figure 2B lower) but significantly weaker than those for matched DNA, consistent with the results for heterogeneous probe particle optical condensation, in which the target DNA molecules were selectively trapped and condensed by laser irradiation. The fluorescence intensity for matched DNA with the homogeneous probe particles was 66% higher than that with the heterogeneous probe particles. This difference is discussed in the next section. Additionally, we examined optical condensation using another homogeneous probe particle comprising a mixture of two DNA-modified AuNP probes (<I> and <II>). In this case, no LIA and condensation of the target DNA molecules were observed at the laser-irradiation area. These results indicated that the LIFs exerted on the AuNPs and target DNA molecules are weak and negligible so the LIFs can not transport the AuNPs and target DNA molecules toward the laser-irradiation area. Since it is experimentally confirmed that the AuNP probe and target DNA molecules are involved in the LIA observed in the heterogeneous probe-particle optical condensation by conducting a supporting experiment with DNA-modified AuNPs and nonmodified PSMPs instead of DNA-modified PSMP (see Figure S2), it is considered that the LIFs exerted on the PSMPs and resulting the nonthermal LIC play an important role in transporting AuNPs and target DNA molecules and the optical condensation of DNA.

### DNA-Sequence Dependence Highlighted with Photothermal Effect

**Figure 3:**
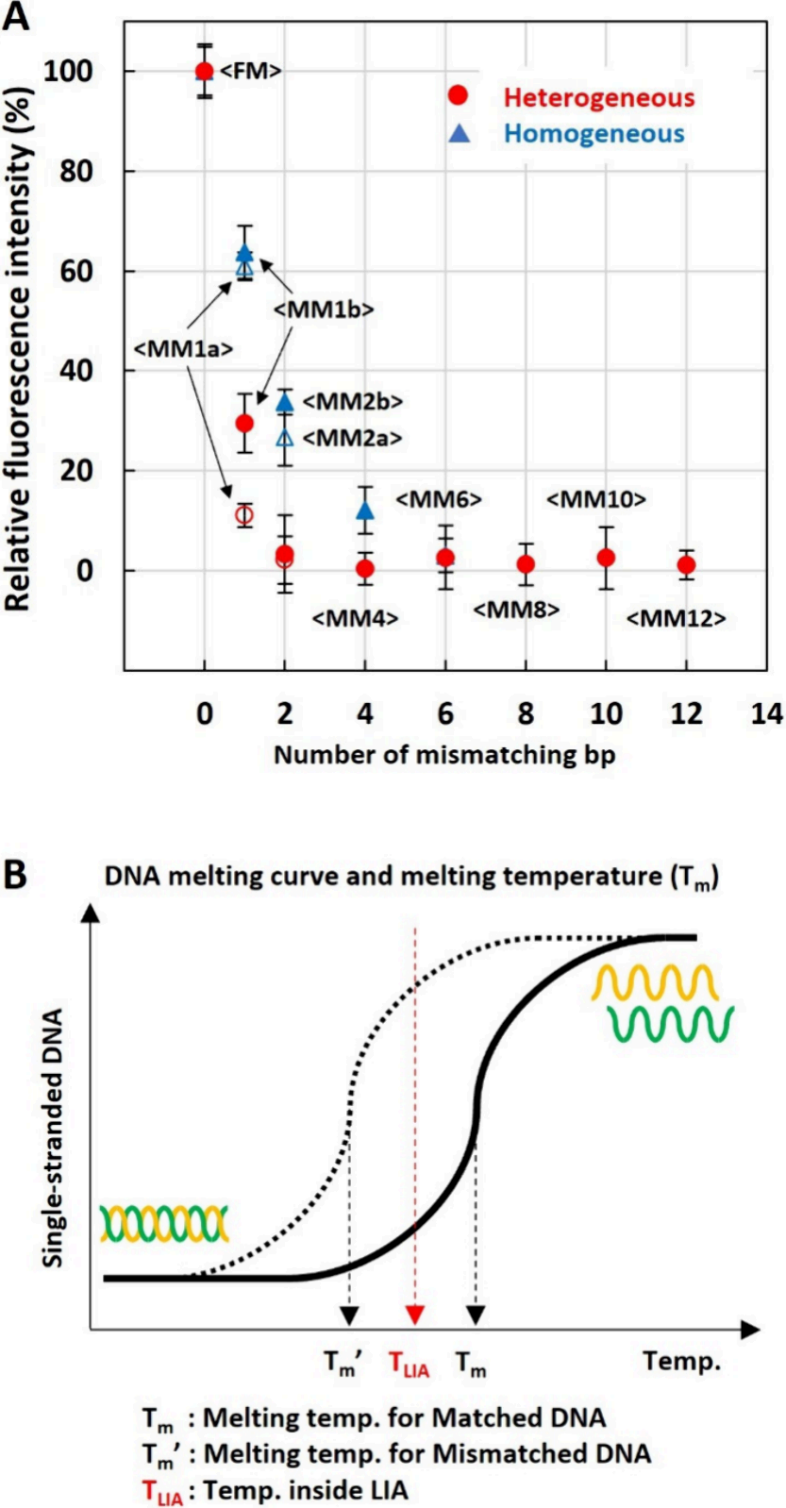
Dependence of optical acceleration on DNA sequence. (A) Relative fluorescence intensity (normalized to that obtained with Matched DNA) with heterogeneous probe particles (red circles) and homogeneous probe particles (blue triangles) with varying numbers of mismatching base pairs. The error bar represents the SD (*n* = 3). For 1-base and 2-base-mismatched DNA, two sequences of each condition (MM1a/MM1b, and MM2a/MM2b) were evaluated. MM1a and MM2a are represented as the opened marker, and MM1b and MM2b are represented as the closed marker. (B) Schematic illustration changes in DNA structure with melting temperature (*T*_m_). The *T*_m_ for Matched DNA is generally higher than that for Mismatched DNA (*T*_m_’). In optical condensation using heterogeneous probe particles, the temperature inside the LIA (T_LIA_) is expected to increase above room temperature owing to the photothermal effect of the AuNPs. See also the DNA sequences in Table S2.

We examined the specificity of optical condensation with hetero- and homogeneous probe particles for the identification of sequence mismatches by detecting target DNA molecules along with complementary (full matched, FM), 1-, 2-, 4-, 6-, 8-, 10-, 12- and 24-base-mismatched (perfect mismatched) DNA (Table S2). We employed two sequences for 1-base and 2-base-mismatched DNA molecules with different mismatched base pairs or sites. Figure 3A shows the relative fluorescence intensities (normalized that obtained with the matched DNA to be 100%) plotted as a function of the number of mismatched bases. In the optical condensation of the heterogeneous probe particles, introduction of one mismatched base reduced the fluorescence intensity by 70–90%, while the second mismatched base further reduced the fluorescence intensity to 0% (comparable to the NC level, Figure 3A). Interestingly, the reduction in fluorescence intensity depended on which base pair was replaced with a mismatched base. In 1-base mismatch 1 (MM1a), in which 2G was replaced with C, the fluorescence intensity was reduced by 90% (opened red circle in Figure 3A for one mismatched base). In 1-base mismatch 2 (MM1b), in which 17A was replaced with T, the fluorescence intensity was reduced by 70% (closed red circle in Figure 3A for one mismatched base). The difference in the reduction in fluorescence intensity can be explained by the number of hydrogen bonds in the base pairs. Three hydrogen bonds were formed in the GC base pair, and two in the AT base pair. Thus, the change in enthalpy was greater in the formation of the GC base pair than that for the AT base pair, and the mismatch in the GC base pair had a larger impact on the total binding energy. In contrast, the fluorescence intensity was reduced only by ∼40% in the optical condensation of homogeneous probe particles with one mismatched base, and no mismatched base pair dependence was observed (opened and closed blue triangles in Figure 3A for one mismatched base). Six mismatched bases were required to reduce the fluorescence intensity to the NC level. These results demonstrated that optical condensation with heterogeneous probe-particles has a higher specificity in the base sequence.

We evaluated the differences in the sensitivity and specificity of hetero- and homogeneous probe-particle optical condensation based on the DNA melting point and photothermal conversion under laser irradiation (Figure 3B). Because DNA hybridization proceeds through hydrogen bonding, binding is weak under high temperatures. DNA molecules tend to exist in double- and single-stranded forms at temperatures lower and higher than the melting temperature (*T*_m_). In a heated environment, DNA hybridization is subject to sequence-specific selection, depending on the binding energy and *T*_m_. Complementary DNA sequences generally have a high *T*_m_ and maintain their double-stranded form even at high temperatures. In contrast, mismatched DNA sequences, which generally have a lower *T*_m_’ than *T*_m_, change to single-strand forms. Therefore, complementary DNA sequences selectively form duplex DNA at a temperature higher than *T*_m_’ but lower than *T*_m_. A heating strategy for a higher base sequence specificity has been employed in the PCR annealing process. Because AuNPs are good plasmonic heat sources,^42−44^ the temperature inside the LIA (T_LIA_) is expected to increase above room temperature under laser irradiation with heterogeneous probe particles, thereby facilitating the selective progression of DNA hybridization by complementary DNA. In addition, it is assumed that the inhomogeneous temperature distribution induced by the laser irradiation and the plasmonic heating with AuNPs would result in faster diffusion and thermophoretic motion toward the outside of the LIA. This thermal effect on mobility is expected to be more prominent on smaller particles and molecules. Thus, there would be two competitive factors; on one hand, AuNPs and target DNA molecules are ejected from the LIA by thermal mobility, and on the other hand, AuNPs and target DNA molecules are captured in the LIA by DNA hybridization. For the matched DNA case, the DNA hybridization may overcome thermal mobility, while for the mismatched DNA case, thermal mobility exclusively ejects unbound AuNPs and target DNA molecules. For these reasons, higher sequence specificity was observed with heterogeneous probe particles than with homogeneous probe particles, in which the AuNPs act as a medium for photothermal conversion. This is consistent with the occasional bubble formation following optical condensation performed with heterogeneous probe particles and a higher laser power (∼800 mW; a typical transmission image is shown in Figure S3). High temperatures may reduce the sensitivity of DNA detection because double-stranded DNA may melt to single-stranded DNA at ∼ T_LIA_. We also theoretically confirmed that light-induced heat proportional to the absorption cross-section of each AuNP^45^ can be greatly enhanced at 1064 nm as the wavelength of irradiated laser because of the spectral redshift via attractive dipole-interaction of LSPs and the broadening via plasmonic super-radiance arising from the quantum effect of interacting LSPs^46,47^ when AuNPs were densely assembled (Figure S4). In contrast, homogeneous probe particles without AuNPs merely increased the temperatures inside the LIA above room temperature, and allowed less thermal mobility. Thus, most of the target DNA molecules even with a mismatched sequence remained as double-stranded DNA or remained in the LIA under laser irradiation, resulting in less specificity but higher sensitivity with homogeneous probe particles.

The high gene-specific DNA detection with the heterogeneous probe particles potentially enables us to distinguish SNPs in DNA by appropriately designing probe DNA sequences. SNPs are key genetic mutations that frequently seen in many diseases like cancer. For example, the KRAS gene, which is related to cell proliferation, has frequently observed SNPs at Codon 12, such as G12C, G12D, and G12 V.^48^ Selectively detecting those SNPs from wild genes in cfDNA containing ctDNA may lead to early cancer diagnosis and prevention of recurrence. There are also recent reports that not only the detection of ctDNA from blood (serum and plasma) but also the analysis of ctDNA from nonblood sources (urine, cerebrospinal fluid, pleural or peritoneal fluid, saliva etc.) would provide a more sensitive information for particular tumor types or locations in patient body.^16^ Additionally, in the emerging field of environmental DNA detection, SNPs are being investigated for species identification under climate change, pollution, and ecosystem changes. The DNA detection using optical condensation with the heterogeneous probe particles experimentally demonstrated in this work exhibits a potential for opening new rapid and highly sensitive and gene-specific detection SNPs, offering early cancer diagnosis, recurrence prevention, and environmental surveys.

### Fluorescence Intensity Depends on DNA Concentration

**Figure 4:**
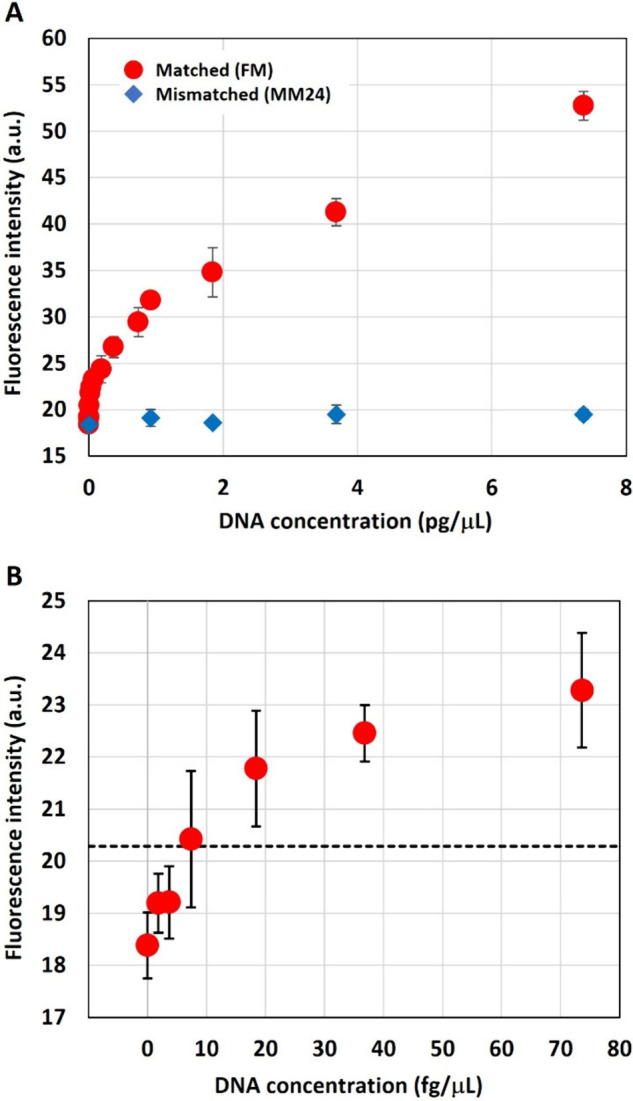
Calibration curve of DNA optical condensation with heterogeneous probe particles. (A) Fluorescence intensities obtained with Matched DNA (red circles) and Mismatched DNA (blue diamonds) were plotted as a function of DNA concentration. The error bars represent the SD (*n* = 3). (B) Closed plot of fluorescence intensity as a function of DNA concentrations ranging from 0 to 80 fg/μL. The dashed line represents the averaged fluorescence intensity +3S.D at the NC (DNA concentration 0 fg/μL).

Finally, we demonstrated that the optical condensation technique can be applied for highly sensitive and quantitative DNA detection without any amplification process (PCR-free). Figure 4A shows the fluorescence intensities obtained using the heterogeneous probe-particles as a function of the target DNA concentration for matched DNA (red circles) and mismatched DNA (blue diamonds). We observed a strong positive correlation between fluorescence intensity and target-DNA concentrations for matched DNA, while fluorescence intensities for mismatched DNA remained at the NC level, even at concentrations of several thousand fg/μL, guaranteeing high specificity. This indicated that the optical condensation technique can effectively detect the concentrations for matched target DNA in a sequence-specific manner.

The obtained fluorescence intensity (i.e., sensitivity of the DNA detection) with the heterogeneous probe-particle optical condensation may be influenced by probe storage conditions, and/or DNA sample conditions, that is temperature, pH, and contamination of other biomolecules. However, it was found that the probes and the techniques were less susceptible to probe storage temperature, and pH level and adding 10 mg/mL of bovine serum albumin (BSA) in the target DNA solution (see Figure S5). The preliminary results promise our technique is applicable for cfDNA/ctDNA detection in a clinical sample or detecting environmental DNA. The exposure time for fluorescence imaging and the photostability of the fluorescent dye (Alexa Fluor 488) may also influence the sensitivity of the detection. We examined the photostability of the dye by measuring 5 consecutive fluorescence images after an optical condensation. As a result, the dye photobleached about 12% within 1 min of light illumination for the fluorescence imaging (see Figure S6). However, the reliability of the detection is ensured by using a constant exposure time and illumination intensity.

Figure 4B shows a closed plot where DNA concentration ranges from 0 to 80 fg/μL. The limit of detection (LOD) was estimated as the first concentration where the value was over the averaged fluorescence intensity plus 3S.D. for NC. The LOD for matched DNA was 7.37 fg/μL, corresponding to 2.36 amol per well. Based on the initial concentration of the target DNA solution, an estimated amount of target DNA was approximately 2.36 amol in the homemade microwell (the volume ∼7.07 μL, 3 mm diameter and 1 mm thickness) and 2.36 ymol in the photometric area (assuming the volume of the LIA, 30 μm diameter and 10 μm thickness). Therefore, we estimated the number of target DNA molecules captured in the LIA would be between 2.36 ymol (in minimum by assuming no DNA condensation at LIA) and 2.36 amol (in maximum by assuming all molecules in the microwell condensed in the LIA). Based on the LOD, the sensitivity of our PCR-free optical condensation technique with only 5 min of laser irradiation was one-order magnitude higher than that for digital PCR (∼200 fg/μL LOD).

## Conclusions

This study applies the “Light-induced Acceleration of Molecular Recognition” strategy to directly generate DNA-modified dissipative structures with heterogeneous probe particles (PSMPs and AuNPs) at the solid–liquid interface with enhanced optical force and photothermal effects without any genetic amplification step (PCR-free). This platform was DNA sequence-dependent and effectively distinguished one-base-pair mutations with a LOD (7.37 fg/μL) one-order magnitude higher than that for digital PCR, with only a few minutes of laser irradiation. The optically assembled microparticles and nanoparticles contained target dye-modified DNA with an estimated amount between 2.36 ymol (2.36 × 10^–24^ mol) and 2.36 amol (2.36 × 10^–18^ mol). This optical condensation-based technique can be used for rapid, highly sensitive, and sequence-specific DNA detection and provides a basis for the high-throughput quantitative measurement of cfDNA, mutation detection in ctDNA, and early diagnosis of diseases with higher specificity when combined with suitable biomarkers. The simplicity, rapid turnover, sensitivity, and specificity of optical condensation with heterogeneous probes may be extendable to other essential molecular recognition processes in addition to DNA hybridization, such as antigen–antibody reactions, sugar-lectin binding, and cell control on small biochips. The underlying mechanism would be applied to elucidate SNPs in DNA, such as KRAS and EGFR,^9,10^ and the quantum probabilistic process of gene mutation.^11^ Overall, our findings will inform the development of a portable platform for detecting environmental DNA in the fields of wildlife conservation and ecosystem management and promote a paradigm shift in liquid biopsies for rapid diagnostics and precision medicine.

## Methods

### Materials

All the chemicals used in this study were of reagent grade. Chloroauric acid (HAuCl_4_, product # 073–00933), trisodium citrate dihydrate (product # 199–01781), and NaCl (product # 191–01665) were purchased from Fujifilm Wako Pure Chemical Industries, Ltd., and were used without any purification. Artificially synthesized oligonucleotides, including 5′-Alexa Fluor 488-capped DNA, 3′- and 5′-terminally thiolated DNA, and 3′- and 5′-terminally biotinylated DNA, were obtained from Thermo Fisher Scientific, and were used without any purification. The used sequences are represented in Table S1. Streptavidin-coated polystyrene microparticle (SA-PSMP) with a diameter of 2 μm (Cat # 24160–1) was purchased from Polysciences Inc. and used without any purification. Coverglass (22 × 32 mm, thickness 0.13–0.17 mm, product # C022321) was purchased from Matsunami Glass Ind., Ltd. Milli-Q grade (>18 MΩ) water with ultraviolet sterilization was used after filtering with 0.2 μm pore size throughout the experiment.

### Preparation of Gold Nanoparticles

AuNPs with an average diameter of 30 nm and a typical concentration of 5.18 × 10^11^ particles/mL were prepared by the citrate reduction of HAuCl_4_. An aqueous solution of trisodium citrate dihydrate (2 wt %, 561 μL) was brought to 25 mL of MQ grade water with a stirrer bar, and heated to 80 °C with stirring (1500 rpm) in a water bath. An aqueous solution of HAuCl_4_ (1 wt %, 750 μL) was quickly added to the heated trisodium citrate solution. The stirring was continued for 20 min at 80 °C, then the solution was removed from the water bath. The solution was allowed to cool to room temperature. The resulting solution was characterized by measuring the UV–vis absorption spectrum with a spectrophotometer (UV-630BIO, JASCO), typically showing an absorption maximum at 526 nm (See a typical absorption spectrum in Figure S7). The derived AuNP dispersion liquid was stored in a glass bottle maintained at 25 °C before the modification of an oligonucleotide.

### Modification of Probe DNA on the Probe Particles

For the modification of oligonucleotide to AuNPs,^33,35,38,39^ 10 μL of 361 μM thiolated oligonucleotide aqueous solution (3′- or 5′-terminally thiolated DNA) was added in 850 μL of a 30 nm AuNP dispersion liquid, and incubated at 25 °C for 24 h. Thereafter, 40 μL of 2.5 M NaCl aqueous solution and 100 μL of 100 mM phosphate buffer (pH = 7.0, product # 168–27155) were added, resulting in 10 mM of phosphate buffer (pH 7.0) and 0.1 M of NaCl. The resulting dispersion liquid was maintained at 25 °C for 40 h, followed by centrifugation (SORVALL Biofuge primo R, Thermo Scientific) for 25 min at 15,000 rpm at ∼5 °C to remove any unreacted oligonucleotide. After the removal of the 950 μL supernatant, the AuNPs were washed with 10 mM phosphate buffer solution containing 0.1 M NaCl. After another round of centrifugation under the same conditions, the precipitate was dispersed in 10 mM phosphate buffer containing 0.3 M NaCl. The AuNPs modified with the 3′- and 5′-terminally thiolated DNA were denoted as Probe <I> and Probe <II>, respectively, as described in Table S1. The surface density of the DNA on a probe AuNP was estimated as 48 pmol/cm^2^.^39^

For the modification of oligonucleotide to SA-PSMPs, 10 μL of 361 μM biotinylated oligonucleotide aqueous solution (3′- or 5′-terminally biotinylated DNA) was added in 470 μL of a 2 μm SA-PSMP dispersion liquid (after 3times washed and 6 times dilution of the original SA-PSMP dispersion liquid with 10 mM phosphate buffer containing 0.1 M NaCl and 10 mg/mL Bovin Serum Albumin), and incubated at 4 °C for 1 h, followed by centrifugation (MCF-1350, LMS) for 3 min at 7.5 kG at ∼25 °C to remove any unreacted oligonucleotide. After the removal of the 456 μL supernatant, the SA-PSMPs were washed with 10 mM phosphate buffer solution containing 0.1 M NaCl. After another round of centrifugation under the same conditions, the precipitate was dispersed in 10 mM phosphate buffer containing 0.3 M NaCl. The SA-PSMPs modified with the 3′- and 5′-terminally biotinylated DNA were denoted as Probe <III> and Probe <IV>, respectively, as described in Table S1. The surface density of the probe DNA on a probe PSMP was estimated as 1.21 pmol/cm^2^. For the estimation, UV–vis absorption spectrum measurements by a spectrometer (V-630BIO, JASCO Corporation) were performed, where three dispersion liquid samples were measured; a) supernatant from the probe DNA-modified PSMP dispersion liquid, b) probe DNA solution, c) supernatant from a SA-PSMP dispersion liquid (6 times diluted from the original). The spectrum c was measured for removing any unwanted additions in spectrum a (for example, surfactants, stabilizers, etc. that are contained in the original SA-PSMP dispersion liquid, and phosphate buffer). Figure S8A shows the obtained three spectra, and Figure S8B shows the difference spectrum (a–c) and spectrum b. An absorbance reduction by the Streptavidin–Biotin binding on the SA-PSMPs was observed in Figure S8B. Absorbance at 260 nm (A_260_) of (a - c) and b were determined as 0.387 and 0.396, respectively. By considering the initial concentration of the probe DNA (C_DNA_ = 3.61 μM), the solution volume (V = 1000 μL), the concentration (C_MP_ = 5.401 × 10^8^ particles/mL) and the surface area of the SA-PSMP (S_MP_ = 12.566 μm^2^), the surface density of the probe DNA on a probe PSMP was calculated as followed,
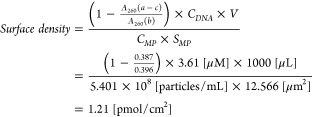


### Light-Induced Acceleration and Fluorescence Imaging

The experimental setup used in this study is schematically described in Figure S9. The continuous infrared laser (ASF1JE01, 1064 nm, Furukawa Electric Co., Ltd.) for optical condensation was guided to an inverted optical microscope (Eclipse Ti–U, Nikon) using a backport adapter (LMSAD-NI-BP; Sigma-Koki). The condensation laser was focused with a 40 × objective lens (CFI S Plan Fluor ELWD 40XC NA = 0.6, Nikon). The input laser power was kept to 640 mW measured after the objective and a coverglass. Ten μL of sample-dispersed mixtures (each 5.0 μL of probe dispersion liquids, for example, 5 μL of Probe <II> and 5 μL of Probe <III>, and 5.0 μL of target DNA in 10 mM phosphate buffer (pH = 7.0)) was encapsulated in a homemade microwell (Figure S10). The homemade microwell consists of two coverglasses and a 1 mm thick spacer (KTD-12, 3 M Japan). A hole was made in the spacer by a cylindrical punch (ϕ ∼ 3 mm). The volume of the homemade microwell is estimated at about 7.07 μL. An optical transmission image of the top coverglass/liquid interface was recorded under bright field conditions using a Complementary Metal-Oxide-Semiconductor (CMOS) camera (DS-Fi3, 2880 × 2048 pixels, Nikon). Then, the 1064 nm laser was irradiated for 5 min, where the laser focus was set to 30 μm above the top coverglass/liquid interface. After the laser irradiation, a transmission image at the top coverglass/liquid interface was recorded again. Thereafter a fluorescence image was also recorded with an excitation light source Hg lamp and a filter set (FITC-A-Basic-NTE, Chroma). The recorded fluorescence images were analyzed with an image analysis software (NIS element, Nikon). Briefly, averaged brightness at the light-induced assembly (LIA) area (40 × 40 pixels at the weight center of LIA) and the background (BG) area (200 × 2048 pixels from the left edge of the image) were determined, and the fluorescence intensity was calculated as the difference of between the averaged brightness at LIA and BG.

## Data Availability

All data are presented in the paper and/or Supporting Information. Additional data may be requested from the corresponding authors.

## Supporting Information Available

Time course of transmission images with the heterogeneous probe-particles and target DNA without laser irradiation, fluorescence intensity for three experimental conditions, optical transmission image of the photothermal effect, theoretically calculated optical absorption of gold nanoparticles, stability, and functionality of probes, confirmation of the photobleaching effect of target DNA, experimentally observed absorption spectrum of AuNPs, surface density of probe DNA, schematic illustrations of experimental setup, DNA sequences, and explanation of the movie (PDF)
Movie of the optical transmission during optical condensation (MP4)
