# Supplementary material for: Single Nucleotide Polymorphism Highlighted via Heterogeneous Light-Induced Dissipative Structure
Source: ACS Sens. 2025 Jan 23;10(2):751–60. doi: 10.1021/acssensors.4c02119 (PMC11877512; doi:10.1021/acssensors.4c02119)
Supplement: Supplementary file 1 — se4c02119_si_001.pdf [file se4c02119_si_001.pdf]

Supporting Information

Single Nucleotide Polymorphism Highlighted via Heterogeneous Light-induced Dissipative Structure

Shuichi Toyouchi[1,2], Seiya Oomachi[1,2,3], Ryoma Hasegawa[1,2,3], Kota Hayashi[1,2,3], Yumiko Takagi[1,2], Mamoru Tamura[1,4], Shiho Tokonami[1,3]*, Takuya Iida[1,2]*

[1]Research Institute for Light-induced Acceleration System (RILACS), Osaka Metropolitan University, 1-2 Gakuencho, Nakaku, Sakai, Osaka 599-8570, Japan.

[2]Department of Physics, Graduate School of Science, Osaka Metropolitan University, 1-2 Gakuencho, Nakaku, Sakai, Osaka 599-8570, Japan.

[3]Department of Materials Science, Graduate School of Engineering, Osaka Metropolitan University, 1-2 Gakuencho, Nakaku, Sakai, Osaka 599-8570, Japan.

[4]Department of Materials Engineering Science, Graduate School of Engineering Science, Osaka University, 1-3 Machikaneyama-cho, Toyonaka, Osaka 560-8531, Japan.

Corresponding authors: t-iida@omu.ac.jp, tokonami@omu.ac.jp

The PDF file includes:

Figs. S1 to S10
Tables S1, S2
Movie S1 caption
References S1, S2

Other Supplementary Materials for this manuscript include the following:

Movies S1

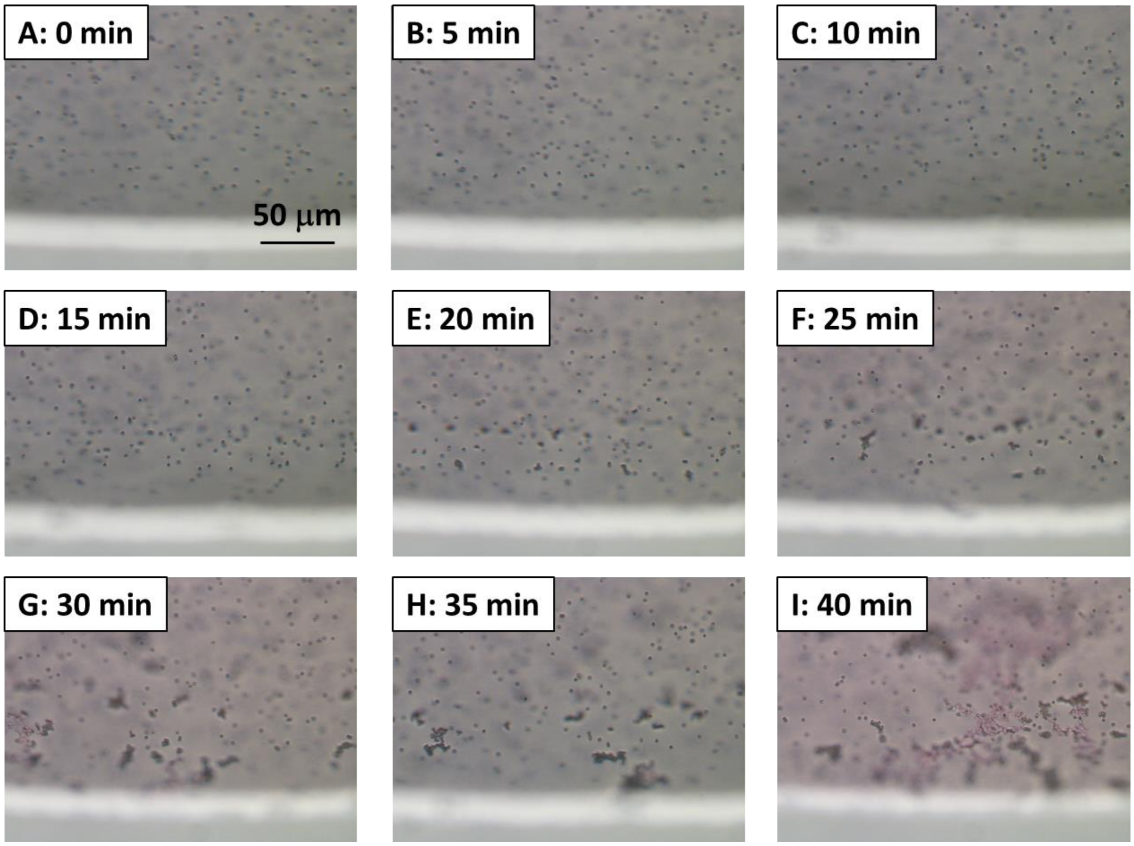

Fig. S1. **The time course of transmission images with the heterogeneous probe and 7.37 ng/μL of target DNA (Matched DNA), but without laser irradiation.** The transmission images were captured at the edge of an unsealed mixture droplet on a cover glass. A spontaneous probe assembly was observed after about 20 min. The scale bar is 50 μm.

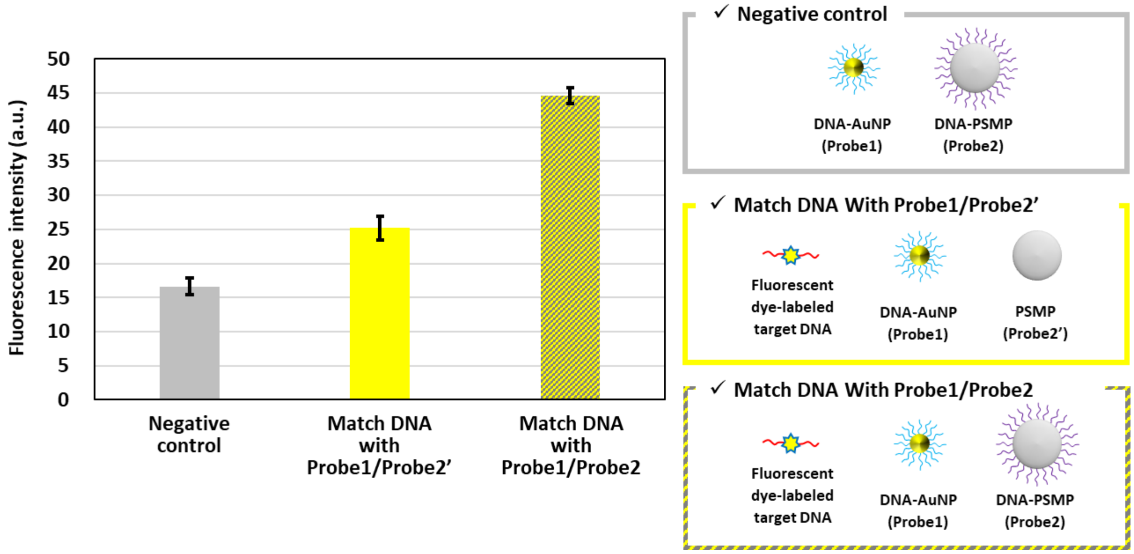

Fig. S2. **Inclusion of AuNPs in the LIA.** (Left) Fluorescence intensity for three experimental conditions, negative control (grey), Matched DNA with Probe1/Probe2’ (yellow), and Matched DNA with Probe1/Probe2 (yellow/grey net hanging). The target DNA concentration was 7.37 pg/μL. (Right) Schematic illustrations of the three conditions used in this experiment. Even without DNA-modified PSMPs, fluorescence intensity at the LIA increased from negative control, indicating that the target DNA molecules were captured and AuNPs were involved in the LIA.

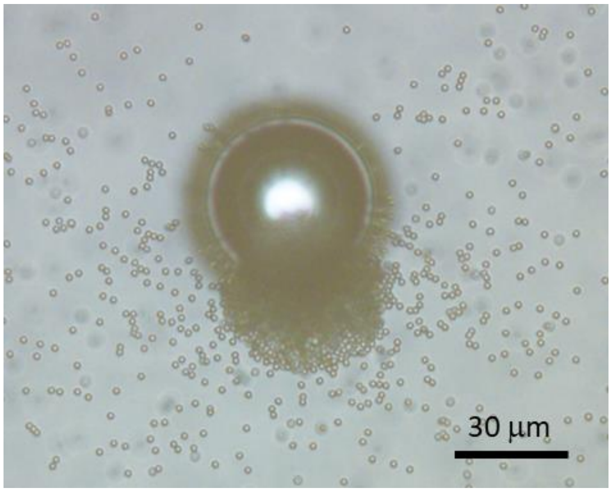

Fig. S3. Photothermal effect. A transmission image recorded after laser irradiation with higher laser power (~800 mW) shows a bubble formation.

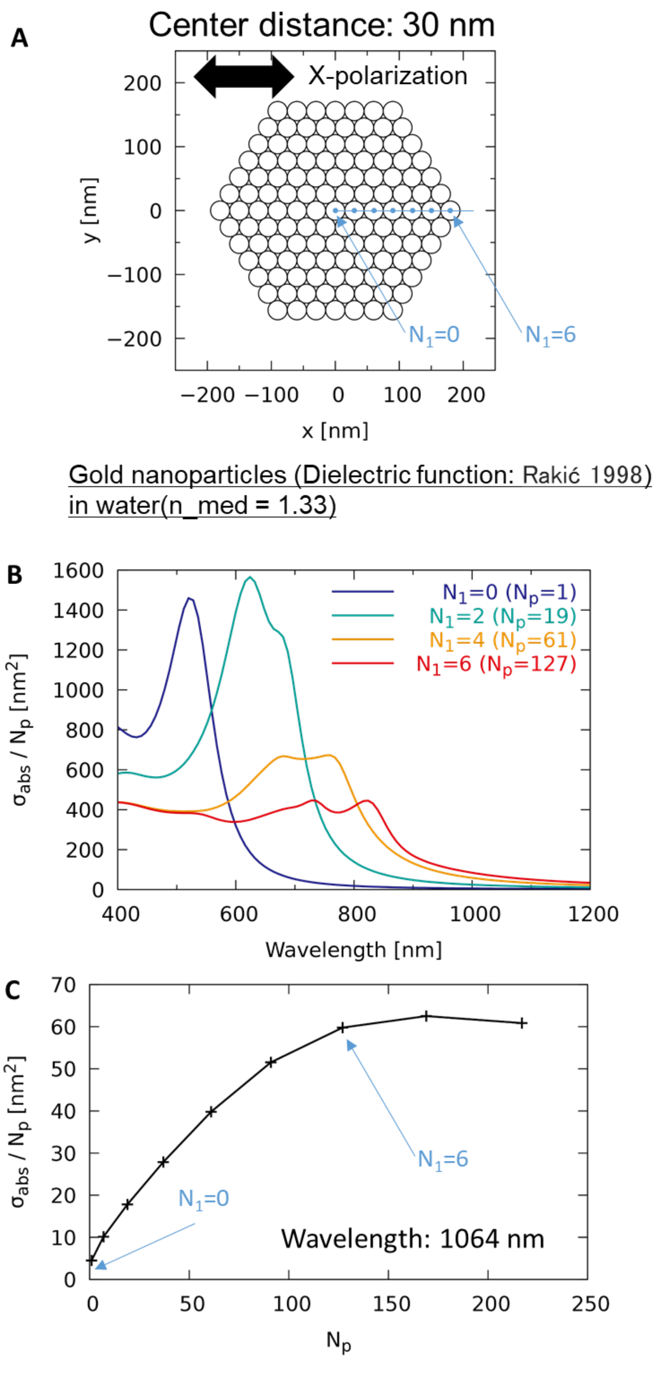

Fig. S4. Optical absorption of assembled gold nanoparticles. (A) Model for the calculation with close-packed gold nanoparticles in triangular lattice. (B) Calculated absorption cross section σ_{abs} of each gold nanoparticle for different number of gold nanoparticles in unit cell N_p in the model (A) using discrete dipole method with spherical cells (DISC method [S1]). Drude-type dielectric function was used for plasmon and Lorentz-type dielectric function were used with the parameters in [S2]. (C) N_p-dependence of σ_{abs} / N_p related with photothermal effect.

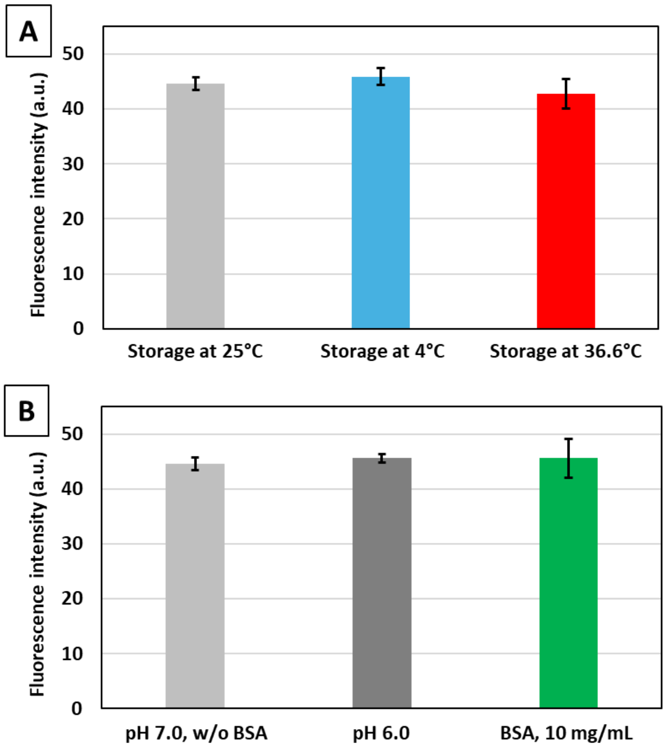

Fig. S5. Influence of the probe conditions on the stability and the functionality. (A) Fluorescence intensity for three probe storage conditions, 25 °C (grey), 4 °C (right blue), and 36.6 °C (red) for 2 weeks. (B) Fluorescence intensity for three target DNA conditions, pH 7.0 and w/o BSA (grey), pH 6.0 (dark grey), and with BSA 10 mg/mL (green). The target DNA (Matched DNA) concentration was 7.37 pg/μL. The error bar is the S.D. (n = 3).

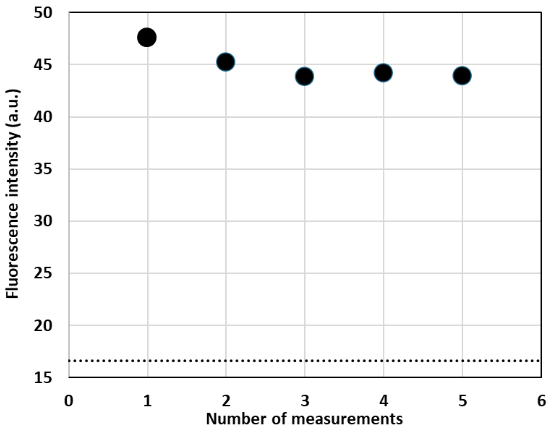

Fig. S6. **Fluorescence intensity over time.** After the optical condensation of 7.37 pg/µL of target DNA (Matched DNA) with the heterogeneous probe, fluorescence imaging was carried out for 5 consecutive times. Each image was obtained with 10 s of exposure time.

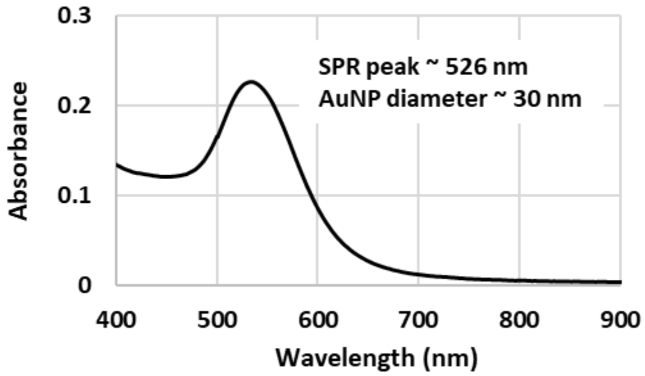

Fig. S7. Absorption spectrum of AuNP solution. The surface plasmon resonance peak is at 526 nm, indicating the AuNP diameter is about 30 nm. The AuNP concentration is estimated to be about 5.18×10^{11} particles/mL.

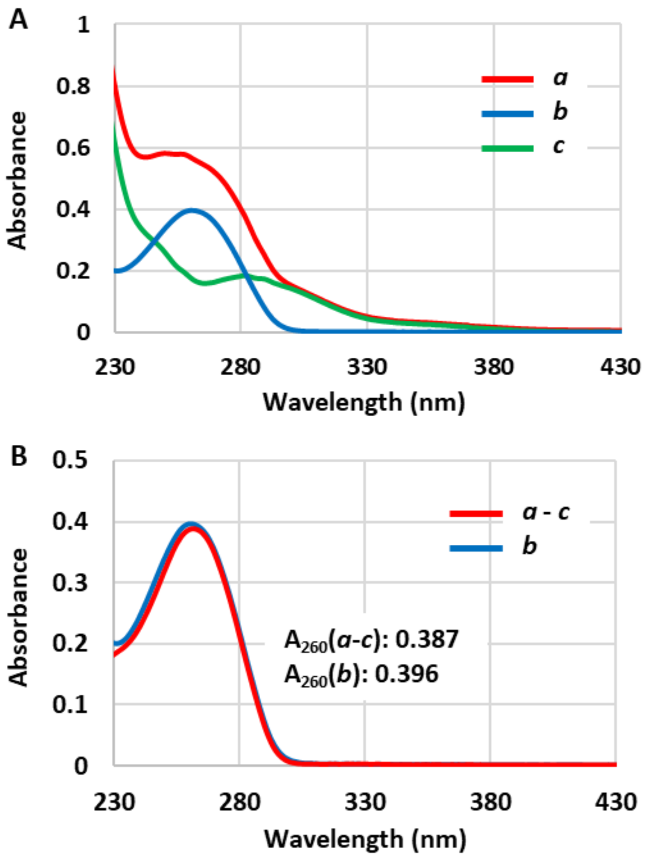

Fig. S8. Estimation of the surface density of probe DNA on a probe PSMP. (A) Three absorption spectra of supernatant from the probe DNA-modified PSMP solution a) with red curve, probe DNA solution b) with blue curve, and supernatant from a SA-PSMP solution c) (6 times diluted from the original) with green curve. (B) Red curve indicates the difference between spectra of a) and c) , and the blue curve indicates the spectrum of b).

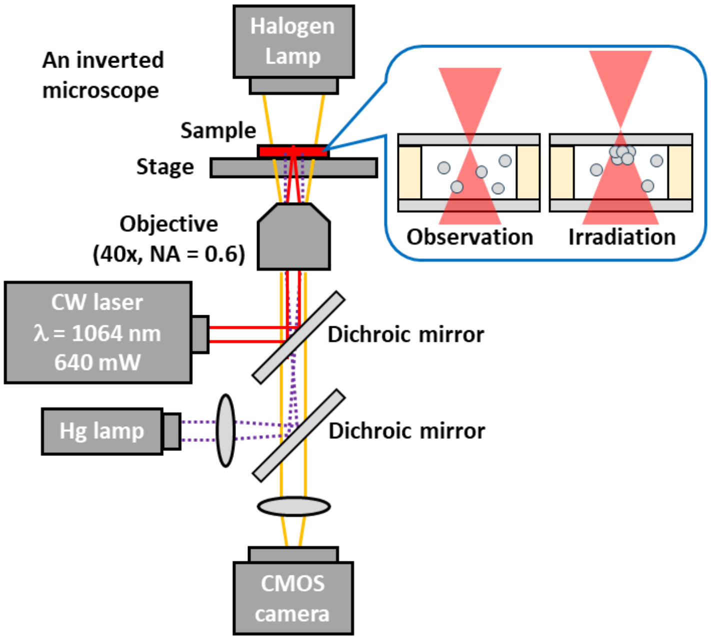

Laser irradiation condition
Microscope: Ti-U (Nikon)
Objective lens: CFI S Plan Fluor ELWD 40x NA = 0.6 (Nikon)
CMOS Camera: DS-Fi3 (Nikon)
CW Laser: ASF1JE01 (Furukawa Electric Co., Ltd.)
Wavelength: 1064 nm
Power: 640 mW
Focus position: 30 μm above (spot size is around 30 μm)
Irradiation time: 5 min

Imaging condition (for transmission)
Record time: 30 ms
Gain: 2.8x
Light source: Halogen lamp
Filter: 1064 cut filter

Imaging condition (for fluorescence)
Record time: 3 s
Gain: 20.9x
Light source: Mercury lamp
Filter: FITC-A-Basic (Ex: 475/30, Em: 530/43)

Fig. S9. Schematic illustration of the experimental setup for the optical condensation.

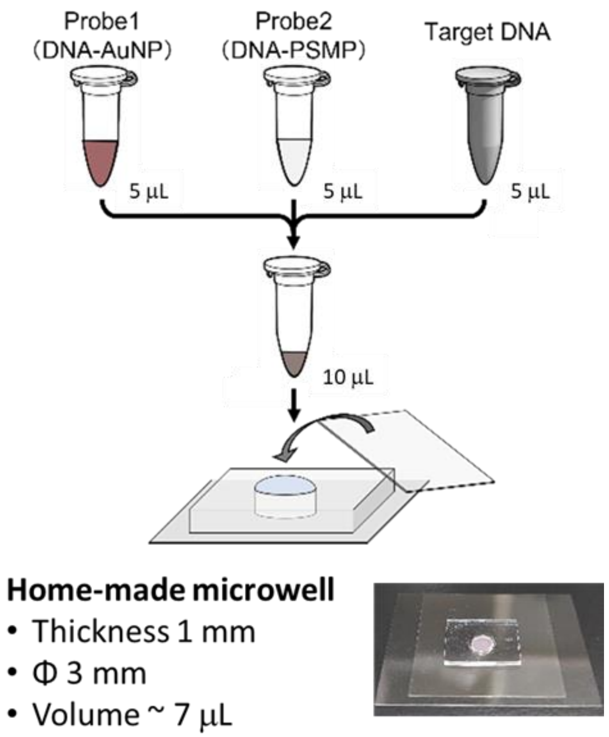

Fig. S10. Schematic illustration of the mixing of probe and target solutions, and the picture of a homemade microwell.

Table S1. Probe-particles and DNA sequences of 12-mer probe DNA.

**Heterogeneous probe-particles (AuNPs-PSMPs)**	**Sequence and modification**
12-mer Probe<I>/12-mer Probe<IV>	5'-SH-$(CH_2)_6$-TCT CAA CTC GTA-3'/5'-ATG CTC AAC TCT-Biotin-3'
**Homogeneous probe-particles (PSMPs-PSMPs)**	
12-mer Probe<III>/12-mer Probe<IV>	5'-Biotin-TCT CAA CTC GTA-3'/5'-ATG CTC AAC TCT-Biotin-3'
**Homogeneous probe-particles (AuNPs-AuNPs)**	
12-mer Probe<I>/12-mer Probe<II>	5'-SH-$(CH_2)_6$-TCT CAA CTC GTA-3'/5'-ATG CTC AAC TCT-$(CH_2)_6$-SH-3'

Table S2. DNA sequences of 24-mer target DNA. Mismatched bases are denoted as bold red characters.

24-mer target DNA	Sequence and modification
**<FM>** Full match	5'-Alexa Fluor 488-AGA GTT GAG CAT TAC GAG TTG AGA-3'
**<MM1a>** 1-base mismatch 1 (2G replaced with C)	5'-Alexa Fluor 488-A**C**A GTT GAG CAT TAC GAG TTG AGA-3'
**<MM1b>** 1-base mismatch 2 (17A replaced with T)	5'-Alexa Fluor 488-AGA GTT GAG CAT TAC G**T**G TTG AGA-3'
**<MM2a>** 2-base mismatch 1 (2G replaced with C, and 19T replaced with A)	5'-Alexa Fluor 488-A**C**A GTT GAG CAT TAC GAG **A**TG AGA-3'
**<MM2b>** 2-base mismatch 2 (10C replaced with G, and 17A replaced with T)	5'-Alexa Fluor 488-AGA GTT GAG **G**AT TAC G**T**G TTG AGA-3'
**<MM4>** 4-base mismatch	5'-Alexa Fluor 488-A**C**A GTT GAG **G**AT TAC G**T**G **A**TG AGA-3'
**<MM6>** 6-base mismatch	5'-Alexa Fluor 488-A**C**A GTT **C**AG **G**AT TAC G**T**G **A**TG AG**T**-3'
**<MM8>** 8-base mismatch	5'-Alexa Fluor 488-A**C**A G**A**T **C**AG **G**AT **A**AC G**T**G **A**TG AG**T**-3'
**<MM10>** 10-base mismatch	5'-Alexa Fluor 488-A**C**A G**A**T **C**AG **GT**T **A**AC G**T**G **A**TG A**CT**-3'
**<MM12>** 12-base mismatch	5'-Alexa Fluor 488-A**CT** G**A**T **C**AG **GT**T **A**AC **CT**G **A**TG A**CT**-3'
**<MM24>** 24-base mismatch (Perfect mismatching)	5'-Alexa Fluor 488-**TCT CAA CTC GTA ATG CTC AAC TCT**-3'

Movie S1. Optical transmission movie during the optical condensation of the heterogeneous probe-particles and target DNA. The 1064 nm CW laser was irradiated on the top coverglass-liquid interface of a homemade microwell containing the heterogeneous probe-particles and target DNA (Match DNA, 7.37 pg/μL). The laser power was 640 mW. The movie is played with 5.5 times speed. When the recording was started, the focal plane of the objective lens was set on the interface. Thus, some probe-particles adsorbing on the coverglass were clearly observed. After the recording was started, the focal plane was intentionally shifted up above 30 μm from the interface. At about 3.6 s later in the movie, the laser irradiation started. After the 5 min laser irradiation (for about 54.5 s in the movie), the focal plane was shifted back on the interface, and an LIA was clearly observed.

References and Notes

S1. Iida, T., Control of Plasmonic Superradiance in Metallic Nanoparticle Assembly by Light-Induced Force and Fluctuations, *J. Phys. Chem. Lett*. **2012**, *3*, 332.

S2. Rakić, A.D.; Djurišić, A.B.; Elazar, J.M.; Majewski, M.L. Optical properties of metallic films for vertical-cavity optoelectronic devices, *Appl. Opt.* **1998**, *37*, 5271.
